# Elevated Numbers of HIV-Specific Poly-Functional CD8^+^ T Cells With Stem Cell-Like and Follicular Homing Phenotypes in HIV-Exposed Seronegative Individuals

**DOI:** 10.3389/fimmu.2021.638144

**Published:** 2021-03-15

**Authors:** Sivasankaran Munusamy Ponnan, Kannan Thiruvengadam, Sujitha Kathirvel, Janani Shankar, Akshaya Rajaraman, Manikannan Mathaiyan, Thongadi Ramesh Dinesha, Selvamuthu Poongulali, Shanmugam Saravanan, Kailapuri Gangatharan Murugavel, Soumya Swaminathan, Srikanth Prasad Tripathy, Ujjwal Neogi, Vijayakumar Velu, Luke Elizabeth Hanna

**Affiliations:** ^1^National Institute for Research in Tuberculosis (Indian Council of Medical Research), Chennai, India; ^2^Centre for Infectious Disease Research, Indian Institute of Science (IISc), Bangalore, India; ^3^Y. R. Gaitonde (YRG) Center for AIDS Research and Education, Chennai, India; ^4^Chennai Antiviral Research and Treatment Centre and Clinical Research Site (CART CRS), Infectious Diseases Medical Center, Voluntary Health Services (VHS), Chennai, India; ^5^Division of Clinical Microbiology, Karolinska Institute, Stockholm, Sweden; ^6^Division of Microbiology and Immunology, Emory Vaccine Center, Yerkes National Primate Research Center, Emory University, Atlanta, GA, United States; ^7^Department of Pathology and Laboratory Medicine, Emory School of Medicine, Emory University, Atlanta, GA, United States

**Keywords:** HIV, HESN, ART, CTL, CXCR5^+^ CD8^+^, TSCM cells

## Abstract

HIV-specific CD8^+^ T cells are known to play a key role in viral control during acute and chronic HIV infection. Although many studies have demonstrated the importance of HIV-specific CD8^+^ T cells in viral control, its correlation with protection against HIV infection remains incompletely understood. To better understand the nature of the immune response that contributes to the early control of HIV infection, we analyzed the phenotype, distribution and function of anti-viral CD8^+^ T cells in a cohort of HIV-exposed seronegative (HESN) women, and compared them with healthy controls and HIV-infected individuals. Further, we evaluated the *in vitro* viral inhibition activity of CD8^+^ T cells against diverse HIV-1 strains. We found that the HESN group had significantly higher levels of CD8^+^ T cells that express T-stem cell-like (TSCM) and follicular homing (CXCR5^+^) phenotype with more effector like characteristics as compared to healthy controls. Further, we observed that the HESN population had a higher frequency of HIV-specific poly-functional CD8^+^ T cells with robust *in vitro* virus inhibiting capacity against different clades of HIV. Overall, our results demonstrate that the HESN population has elevated levels of HIV-specific poly-functional CD8^+^ T cells with robust virus inhibiting ability and express elevated levels of markers pertaining to TSCM and follicular homing phenotype. These results demonstrate that future vaccine and therapeutic strategies should focus on eliciting these critical CD8^+^ T cell subsets.

## Introduction

The World Health Organization (WHO) estimates that there are currently about 33 million people living with the human immunodeficiency virus (HIV) (1). Antiretroviral therapy (ART) is very effective at suppressing HIV replication and prolonging infected individuals' lives by reducing viral load to undetectable levels in the plasma. However, infected individuals need to be on ART for the rest of their life due to persisting viral reservoirs in the body (2–5) that lead to rapid viral rebound following treatment interruption. In order to make progress toward achieving sustained viral remission, complementary approaches that can reduce latent HIV-1 reservoirs and enhance anti-HIV immunity in drug-treated individuals are needed (6).

The hallmark of the adaptive immune response is the generation of long-lived immunological memory following exposure to an antigen (7). Several studies have demonstrated the significance of antiviral CD8^+^ T cells in the control of HIV infection. Proof for the central role of CD8^+^ T cells in the control of HIV infection has been provided through the demonstration of viral re-emergence upon CD8^+^ T cell depletion *in vivo* in SIV-infected rhesus monkeys (RM) that control viremia (8). CD8^+^ T cells have been shown to be important for controlling virus replication even during suppressive ART (9). More recent studies have shown that HIV-specific CD8^+^ T cells with unique phenotypic characteristics, such as CXCR5^+^ CD8^+^ T cells and TSCM cells, are associated with natural control of HIV and Simian Immunodeficiency Virus (SIV) infection (10–15). Contemporary vaccine strategies designed to elicit high frequencies of anti-viral CD8^+^ T cells have used pathogenic SHIV (16) and SIV challenges in rhesus macaques (17–21). In addition to the magnitude, the breadth of the T cell response is also critical for the control of HIV infection, given the enormous capacity of the virus to mutate (18). Studies in humans have demonstrated that both the function and the frequency of anti-viral CD8^+^ T cells and NK cells (22–25) are crucial for viral control (26). Thus, therapeutic interventions against HIV should aim to improve both the magnitude and function of anti-viral CD8^+^ T cells.

HIV resistance has been studied in different contexts within humans (27). Initial reports described exposed yet uninfected individuals, infants exposed to HIV in utero but remained uninfected, HIV negative partners of HIV-infected individuals (serodiscordant couples) and those have had unprotected sexual contact with infected partners such as commercial sex workers and have remained seronegative (28, 29). These exposed seronegative (HESN) individuals were found to have elevated CTL responses to HIV (30). Host-genetic and viral factors are both thought to play a role in the non-progression of disease (31). Certain innate immune factors have also been implicated in viral control in HESN individuals, but the features of antiviral immunity that result in effective virus control are only partially understood. Hence, understanding the role of immune cells and the interaction between HIV and the immune system in HESN individuals would shed more light on the correlates of protection against HIV infection and suggest strategies that could contribute to the development of a functional cure for HIV.

In the present study, we sought to characterize the antiviral CD8^+^ T cell response in a group of naturally resistant HESN individuals and understand its role in protecting against HIV infection and disease progression. We show that HESN individuals have elevated levels of anti-viral CD8^+^ T cells that are phenotypically unique and express high levels of markers pertaining to T stem cell likeness as well as follicular homing phenotype, and express CXCR5 on their surface. In addition, we demonstrate that the HESN population has a higher frequency of HIV-specific poly-functional CD8^+^ T cells with robust virus inhibiting ability. Thus, the study sheds light on some of the critical CD8^+^ T cell subsets and immune responses that a protective HIV vaccine should elicit.

## Materials and Methods

### Ethics Statement

The study protocol was approved by the Scientific Advisory Committee of the ICMR-National Institute for Research in Tuberculosis (NIRT), Chennai, India. The study was conducted in accordance with Good Clinical Laboratory Practice (GCLP) guidelines. The protocol was reviewed and approved by the Institutional Ethics Committee of ICMR-NIRT (IEC ID-2015015) and the Institutional Review Board of the Y. R. Gaitonde Centre for AIDS Research and Education (YRG CARE; YRG-302), Chennai, India.

### Study Participants

The study was carried out at ICMR-NIRT, and specimens were collected at YRG CARE, one of the largest tertiary referral HIV Care Centres in southern India, providing medical care and support for more than 22,000 patients. The study population consisted of 4 groups of individuals: (1) Seronegative female spouses of HIV-1 seropositive men (HIV discordant couples) (HESN; *n* = 35); (2) HIV-unexposed uninfected healthy women (HU; *n* = 35); (3) HIV-infected women on ART (HIV^+^ART^+^; *n* = 10), and (4) HIV-infected women naïve to ART (HIV^+^ART^−^; *n* = 10). The HIV status of the HESN individuals was confirmed by serology as well as PCR. All except 2 of the partners of the HESN individuals had undetectable viral load at the time of recruitment of the HESN persons into the study since they were initiated on ART. This was due to the implementation of the Treat All policy as per the National Technical Guidelines for Antiretroviral Therapy (2017) where all HIV-infected individuals were started on ART irrespective of their CD4 count or clinical stage of disease. However, all couples had been in sexual contact for 3 years or more prior to this and had continued to have unprotected sex post-treatment initiation. ART was given as per the National Technical guidelines on Antiretroviral Treatment (NACO, May 2013). Enrolment to the study required willingness of participants to provide written informed consent for specimen collection and storage. Enrolment criteria for each cohort were as follows: HIV-exposed seronegative women were recruited if women were aged between 20 and 35 years, were seronegative for HIV, and had a history of multiple unprotected sex events (more than two) with an HIV-infected partner during the past 1 year. HIV-infected women naïve to ART were recruited if women were aged between 20 and 35 years, were HIV infected, and naïve to antiretroviral treatment. HIV-infected women on ART were recruited if women were aged between 20 and 35 years, were HIV infected and currently receiving antiretroviral treatment. HIV-unexposed healthy women were recruited if women were aged between 20 and 35 years, were seronegative for HIV, with no risk of HIV acquisition. Healthy women were excluded if women were outside the reproductive age group or were on menstruation, had STIs or other major illnesses, moribund individuals, as well as pregnant and lactating women.

### Sociodemographics

No difference was observed between the groups for age, presence of bacterial vaginitis and Chlamydia trachomatis (CT), or Neisseria gonorrhoeae (NG) infection. All study participants were screened for Human papillomavirus (HPV) infection using the Pap smear test and found to be negative. The HESN group reported sex on an average of about two times per month with their infected partner, with or without condom use. Samples were collected at least a week after the previous sexual act and 2 weeks after the start of the previous menstrual period (Table 1).

**Table 1:** Demographic characteristics of the study population at enrollment.

	**HESN**	**HU**	**HIV^**+**^ ART^**+**^**	**HIV^**+**^ ART^**−**^**
	***N =* 37**	***N =* 35**	***N =* 10**	***N =* 10**
Age mean (range)	36 (27-42)	33 (22-42)	34 (32-39)	35 (30-48)
Cervical Cancer (HPV)	0	0	0	0
STDs (BV, CT, NG)	0	0	0	0
HIV infected partner on ART	37 (100%)	0	NA	NA
Viral Load, Log_10_ copies/mL, mean (SD)	NA	NA	2.14	4.4943 (0.9036)
CD4 Count at treatment initiation (cells/mL), median (IQR)	NA	NA	392 (289–492)	NA

*HPV, Human Papilloma Virus; BV, Bacterial Vaginitis; CT, Chlamydia trachomatis; NG, Neisseria gonorrhoeae; HESN, HIV exposed seronegative; HIV^+^ART^+^, HIV-infected women on ART; HIV^+^ART^−^, HIV-infected women naïve to ART; HU, HIV unexposed seronegative controls*.

### Isolation and Cryopreservation of PBMC

Ten milliliters of blood was collected from all study participants by venepuncture in a green top VACUTAINER® containing sodium heparin as the anticoagulant and used for the isolation of peripheral blood mononuclear cells (PBMC). PBMC were isolated by density gradient centrifugation and cryopreserved at < −190°C as described previously (32).

### Measurement of Soluble Markers in Plasma Specimens Using Cytometric Bead Array

Levels of pro-inflammatory cytokines/chemokines (IL-8, IP-10, EOTAXIN, TARC, MCP-1, RANTES, MIP-1α, MIG, ENA-78, MIP-3α, GRO-α, I-TAC, MIP-1β) were estimated in plasma using the Biolegend LEGEND plex Human Multi-analytic cytometric bead array (CBA; Bio legend, San Diego, CA, USA) following the manufacturer's recommendation. Briefly, beads coated with 13 specific capture antibodies were mixed. Subsequently, 50 μL of the mixed capture beads, 50 μL of plasma diluted 1:2 or 1:20, and 50 μL of detector antibody were added and incubated for 2 h on a plate shaker (250 rpm) at room temperature in the dark. Fifty microliters of streptavidin-phycoerythrin (SA-PE) detection reagent was added to each assay tube and incubated for 30 min on a plate shaker (~250 rpm) at room temperature in the dark. The samples were washed with 1 mL of wash buffer (at 200 g) for 5 min. The bead pellet was resuspended in 300 μL of wash buffer after discarding the supernatant. Samples were analyzed on a BD FACS ARIA SORP flow cytometer using the LEGENDplex data analysis software v8.0 (Biolegend). Individual cytokine concentrations were indicated by their fluorescence intensities. Cytokine standards were serially diluted to facilitate the construction of calibration curves necessary for determining the protein concentration of the test samples. The theoretical limits of detection of the pro-inflammatory cytokines were 1.4 pg/mL for IL-8, 1.1 pg/mL for IP-10, 1.4 pg/mL for EOTAXIN, 0.8 pg/mL for TARC, 0.9 pg/mL for MCP-1, 4.3 pg/mL for RANTES, 2.1 pg/mL for MIP-1α, 9.4 pg/mL for MIG, 1.1 pg/mL for ENA-78, 2.5 pg/mL for MIP-3α, 6.7 pg/mL for GRO-α, 1.1 pg/mL for I-TAC, and 1.4 pg/mL for MIP-1β.

### Intracellular Cytokine Staining (ICS) Assay

The quantity and functionality of antigen-experienced T cells were assessed by flow cytometry in terms of the frequency and type of cytokines they produced. Archived PBMC specimens were analyzed for surface-antigen and cytokine-secretion, adopting the standard ICS assay protocol in conjunction with polychromatic flow cytometric analysis as described previously (33). Cryopreserved PBMC were thawed and rested overnight. Cells were washed, resuspended at a concentration of 1 × 10^6^ cells in 200 μL of RPMI medium supplemented with 10% FBS and stimulated with 1 μg/ml of anti-CD28/49d monoclonal antibodies and 1.5 μg/mL of HIV peptide pools (Env, Gag, Pol—Supplementary Table 2), or 1 μg/mL staphylococcal enterotoxin B (SEB; Sigma-Aldrich, India) and BD golgistop (Becton, Dickinson, India) for 6 h at 37°C. The stimulated cells were stained with 50 μL of Aqua Amine Reactive Viability Dye (L34962) (Invitrogen, India), CD3 APCH7, CD4 BUV737, CD8^+^ AF700, CD45RO BUV395, CCR7 PECY7, and intracellularly with IFN-γ APC, IL-2 PE and TNF-α FITC (Clone details provided in Supplementary Table 1). A minimum of 500,000 events was acquired on a custom-built BD FACS Aria SORP flow cytometer. The results were analyzed using FlowJo software, v 10.5 (Tree Star Inc., Ashland, Oregon, USA). Cytokine secretion was regarded as positive if values were at least twice that of the controls.

### Expansion of CD4^+^ and CD8^+^ T Cells

PBMC were thawed and resuspended at 2 × 10^6^ cells/ml in RPMI containing 10% Fetal Bovine Serum (FBS) with 50 units of IL-2 (R10/50) and 0.5 μg/ml of CD3^+^/CD4^+^ or CD3^+^/CD8^+^ bispecific antibody for expansion of CD8^+^ and CD4^+^ T cell subpopulations, respectively. Culture volumes were doubled at days 3 and 6 with R10/50 medium. Typical purities of 7-day cultures were 97 and 87% for CD4^+^ and CD8^+^ T cells, respectively, suggesting positive expansion and enrichment (≥90% of the CD3^+^ T cells in culture) of the required T cell sub-population (34).

### Viral Inhibition Assay

Viral inhibition assay (VIA) was performed as described by Spentzou et al. (34). Briefly, separate cultures of 7 day-expanded CD4^+^ T cells were infected with HIV-1 isolates belonging to subtype A (U455), subtype B (III B), subtype C (247FV2), subtype D (CBL4), and subtype AD (ELI), at a multiplicity of infection (MOI) of 0.01. Virus-infected target cells were co-cultured with autologous CD8^+^ effector T cells obtained from each study subject. Every 3–4 days, half of the culture supernatant was removed and assessed for Gag p24 content using a commercially available enzyme-linked immunosorbent assay (ELISA) (PerkinElmer, United Kingdom). CD8^+^ T cell-mediated inhibition was defined as ≥log_10_ reduction in the p24 content of day 13 supernatants from CD8^+^ and CD4^+^ T cell co-cultures as compared to that of CD4^+^ T cells alone. Cut-offs were defined by the 97.5th percentile of the VIA response of baseline samples as estimated using PROC QUANTREG in SAS 9.2 (SAS Institute Inc., Cary, NC). VIA response for each virus was defined as positive if the log_10_ inhibition was > 1.5 for all HIV-1 isolates.

### Viruses and Cell Lines

HIV-1 subtype A (U455), subtype B (IIIB), subtype C (247FV2), subtype D (CBL4), and subtype AD (ELI) were used to assess cross-recognition and inhibition of various HIV-1 clades in the VIA. All viruses were obtained from the NIH AIDS Research and Reference Reagent Program (Bethesda, MD), except 247FV2, which was kindly provided by George Shaw (University of Pennsylvania, PA). The titer of each viral isolate was determined by end-point dilution and defined as the 50% tissue culture infectious dose (TCID_50_). Viral titers were determined using the TZM-bl cell line as described by Spentzou et al. (34). Cell lines were obtained from the NIH AIDS Research and Reference Reagent Program.

### Phenotypic Analysis of Immune Cell Subsets

Cryopreserved PBMC obtained from the study participants were used for this analysis. Two million cells were washed with FACS buffer and stained with Live/Dead Fixable Aqua blue dye (Invitrogen). The following cocktail of monoclonal antibodies was used to enumerate the different cell types (Supplementary Table 1): T follicular helper and cytotoxic cell and Treg panel: CD3-APC H7, CD4-PERCP CY5.5 CD8^+^-BUV737, CD45RO-BUV395, CCR7-PEcy7, CXCR3-APC Alexa 700, CXCR5-BB515, PD-1-PE CD25-APC, CD127-PECF594; B cell panel: CD3-APC H7, CD38-BUV395, CD20-APC, CD19-BUV737, IgD-PE, CD27-BB515, CD95-PECF594; Memory T cell and T_SCM_ panel: CD3-APCH7, CD8^+^-APCR700, CD4-BUV737, CD45RO-BUV395, CCR7-PEcy7, CD45RA-BB515, CD95-PECF594, CD28-APC, CD122-PE. All flowcytometric experiments were carried out with proper FMO controls and gatings were applied accordingly. Cells were stained with the antibodies for 20 min at 4°C (antibody and clone description are provided in Supplementary Table 1). About 2 × 10^6^ cells were stained for each panel. After staining, the cells were washed, fixed with BD Cytofix (2% paraformaldehyde), and analyzed on a FACS ARIA III flow cytometer (Becton Dickinson). A minimum of 1,000,000 total events was acquired, and data were analyzed using FlowJo software, version 10.5 (Tree Star Inc., Ashland, Oregon, USA).

### Statistical Analysis

Statistical analyses were performed using GraphPad Prism, version 7.05 (GraphPad Software Inc., CA). Values are presented as median and interquartile range. Percentage frequency of the immune cell subsets like memory T cells, follicular helper-like CD8^+^ T cells (CXCR5^+^ CD8^+^ T cells), and TSCM cells were compared between the seronegative female spouses of HIV-1 seropositive men (HIV discordant couples) (HESN), HIV-unexposed uninfected healthy women (HU), HIV-infected women on ART (HIV^+^ART^+^), and HIV-infected women naïve to ART (HIV^+^ART^−^) groups using Kruskal-Wallis test followed by subgroup analysis using Dunn's multiple comparison test. Mann-Whitney's *T-*test was used to examine the difference in frequency (%) of poly-functional T cells, levels of pro-inflammatory soluble markers, and VIA response between the HESN and HU groups. For all analyses, differences were considered significant if the *p* value was < 0.05.

## Results

### HESN Individuals Possess Significantly Higher Frequencies of CD8^+^ Effector Memory T Cells

**Figure 1:**
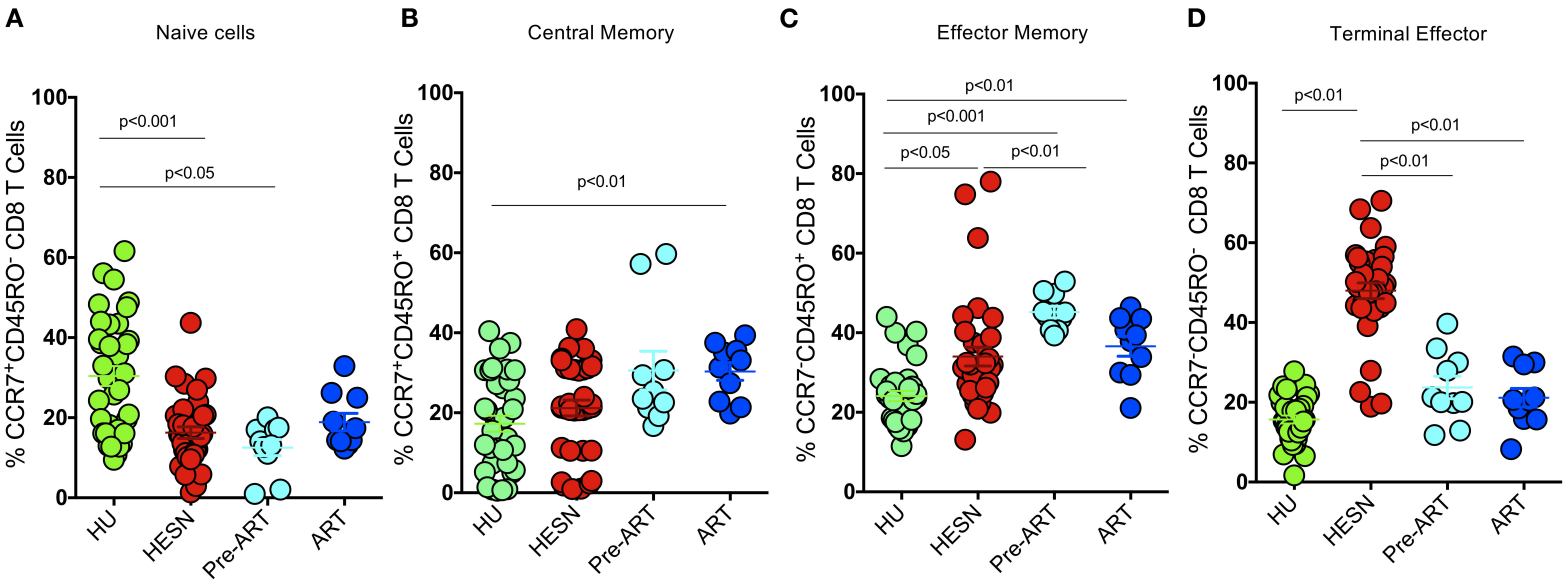
Elevated memory CD8^+^ T cells in HIV-1 exposed seronegative women. Graphical representation of the % frequency of CD8^+^ memory T cell subsets defined using CCR7 and CD45RO in the study groups (HESN—*N* = 35, HIV^+^ART^+^–*N* = 10, HIV^+^ART^−^–*N* = 10, and HU—*N* = 35). **(A)** Data showing the cumulative frequency of CCR7^+^ CD45RO^+^ Central memory CD8^+^ T cells in the HESN, HIV^+^ART^+^, HIV^+^ART^−^ and HU groups. **(B)** Data showing the cumulative frequency of CCR7^−^ CD45RO^+^ Effector memory CD8^+^ T cells in the HESN, HIV^+^ART^+^, HIV^+^ART^−^, and HU groups. **(C)** Data showing the cumulative frequency of CCR7^+^ CD45RO^−^ naïve CD8^+^ T cells in the HESN, HIV^+^ART^+^, HIV^+^ART^−^, and HU groups. **(D)** Figure showing the cumulative frequency of CCR7^−^ CD45RO^−^ Terminal effector memory CD8^+^ T cells in the HESN, HIV^+^ART^+^, HIV^+^ART^−^, and HU groups. The scatter dot plots summarize the % frequency of total memory T cells (median, 1st, and 3rd quartiles). *p-*values were calculated using the K-Wallis test. Sub-group analysis was performed using Dunn's test. (HESN, HIV exposed seronegative; HIV^+^ART^+^, HIV-infected women on ART; HIV^+^ART^−^, HIV-infected women naïve to ART; HU, HIV unexposed seronegative controls).

To define the phenotypic characteristics and distribution of memory CD8^+^ T cell subsets, we performed multicolor flow cytometry to analyze CD45RO and CCR7 expression on CD8^+^ T cells. CD8^+^ Memory T cells were identified as: central memory CD8^+^ T cells (CM) defined as CD3^+^CD4^−^/CD8^+^CD45RO^+^CCR7^+^ cells, effector memory CD8+ T cells (EM) defined as CD3^+^CD4^−^/CD8^+^CD45RO^+^CCR7^−^ cells, terminal effector CD8+ T cells (TE) defined as CD3^+^CD4^−^/CD8^+^CD45RO^−^CCR7^−^ cells and naïve CD8^+^ T cells (TN) defined as CD3^+^CD4^−^/CD8^+^CD45RO^−^CCR7^+^ cells as described by Gattinoni et al. (35) and Mahnke et al. (36). The proportion of CD8^+^ naïve T cells was found to be significantly lower in the HESN group as compared to the healthy control group. The HIV^+^ART^+^ and HIV^+^ART^−^ groups also had lower levels of naïve CD8^+^ T cells as compared to the healthy control group, but the difference between the groups was not significant (Figure 1A). The proportion of CD8^+^ central memory T cells was found to be similar in the HESN and healthy control groups (Figure 1B). Interestingly, in contrast to naïve and central memory CD8^+^ T cells, the effector memory CD8^+^ T cells were significantly higher in the HESN group as compared to the healthy control group. The HIV-infected ART naïve group had the highest proportion of CD8^+^ effector memory T cells [CD8^+^ EM: median of 44.60% (range: 40.70–49.80%) *p* < 0.001] (Figure 1C). The frequency of CD8^+^ TE cells was found to be significantly higher in the HESN group than in the other groups [CD8^+^ TE: median of 48.80%: (range: 44.30–55.30%) *p* = 0.03] (Figure 1D). Taken together, these data demonstrate the presence of a significantly higher proportion of effector memory CD8^+^ T cells in the HESN group implying that they might be the one of the factors that contribute to early control of HIV infection.

### CD8^+^ T Cells in HESN Individuals Express Higher Levels of TSCM and Follicular Homing Markers

**Figure 2:**
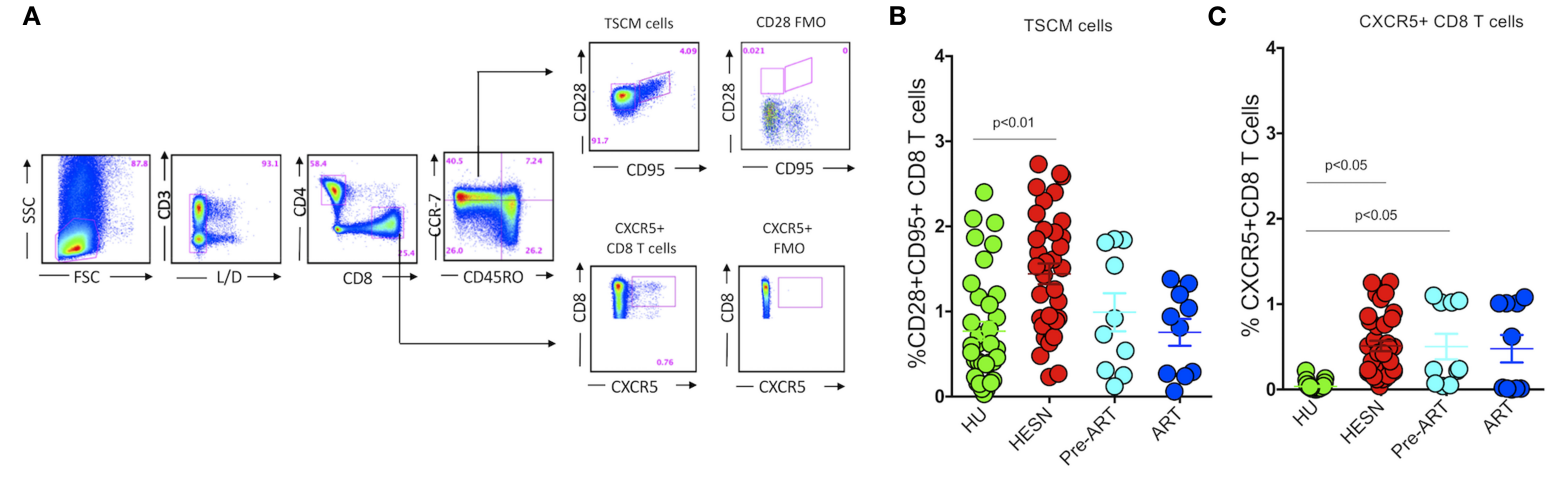
Abundance of CXCR5^+^ CD8^+^ T cells and stem-like memory CD8^+^ T cells in HIV-1 exposed seronegative women. **(A)** Representative flow plots showing the % frequency of CXCR5^+^ T cells and TSCM cells in the study groups (HESN—*N* = 35, HIV^+^ART^+^–*N* = 10, HIV^+^ART^−^–*N* = 10 and HU—*N* = 35). **(B)** Figure showing the cumulative frequency of CCR7^−^CD28^+^CD95^+^ stem cell-like memory subset of CD8^+^ T cells in the HESN, HIV^+^ART^+^, HIV^+^ART^−^ and HU groups. **(C)** Figure showing the cumulative frequency of CXCR5^+^ CD8^+^ T cells in the HESN, HIV^+^ART^+^, HIV^+^ART^−^ and HU groups. The scatter dot plots summarize the % frequency of total CD8^+^ CXCR5^+^ T cells and TSCM cells (median, 1st, and 3rd quartiles). *p-*values were calculated using the K-Wallis test. Sub-group analysis was performed using Dunn's test.

Recent studies reveal that those with controlled HIV infection possess long-lived, self-renewing memory stem cell-like CD8^+^ T cells (TSCM) for the maintenance of functional immunity (37, 38), and suggest that follicular homing CXCR5^+^ CD8^+^ T cells are effectively involved in the control of HIV and SIV infections in animal models (39, 40). We therefore analyzed the phenotype of CD8^+^ T cells in the HESN population and compared it with that seen in the HIV-infected and HU groups. Interestingly, we found that the HESN group had significantly higher numbers of TSCM cells in the CD8^+^ T cell compartment than HIV-infected individuals (Figures 2A,B). We also analyzed the expression of CXCR5 on these cells and found that the frequency of CXCR5^+^ CD8^+^ T cells was significantly higher in the HESN group as compared to the other groups (*p* < 0.05) (Figure 2C). The median frequency of circulating CXCR5^+^ CD8^+^ T cells in the HESN group was 0.42% (range: 0.20–0.76%). Collectively, our findings reveal a significantly expanded population of CXCR5 receptor-expressing T cells and TSCM cells in the HESN group, indicating a plausible role for these cell types in resistance to HIV infection.

### Presence of Strong HIV-Specific Poly-Functional CD8^+^ T Cell Responses in HESN Women

**Figure 3:**
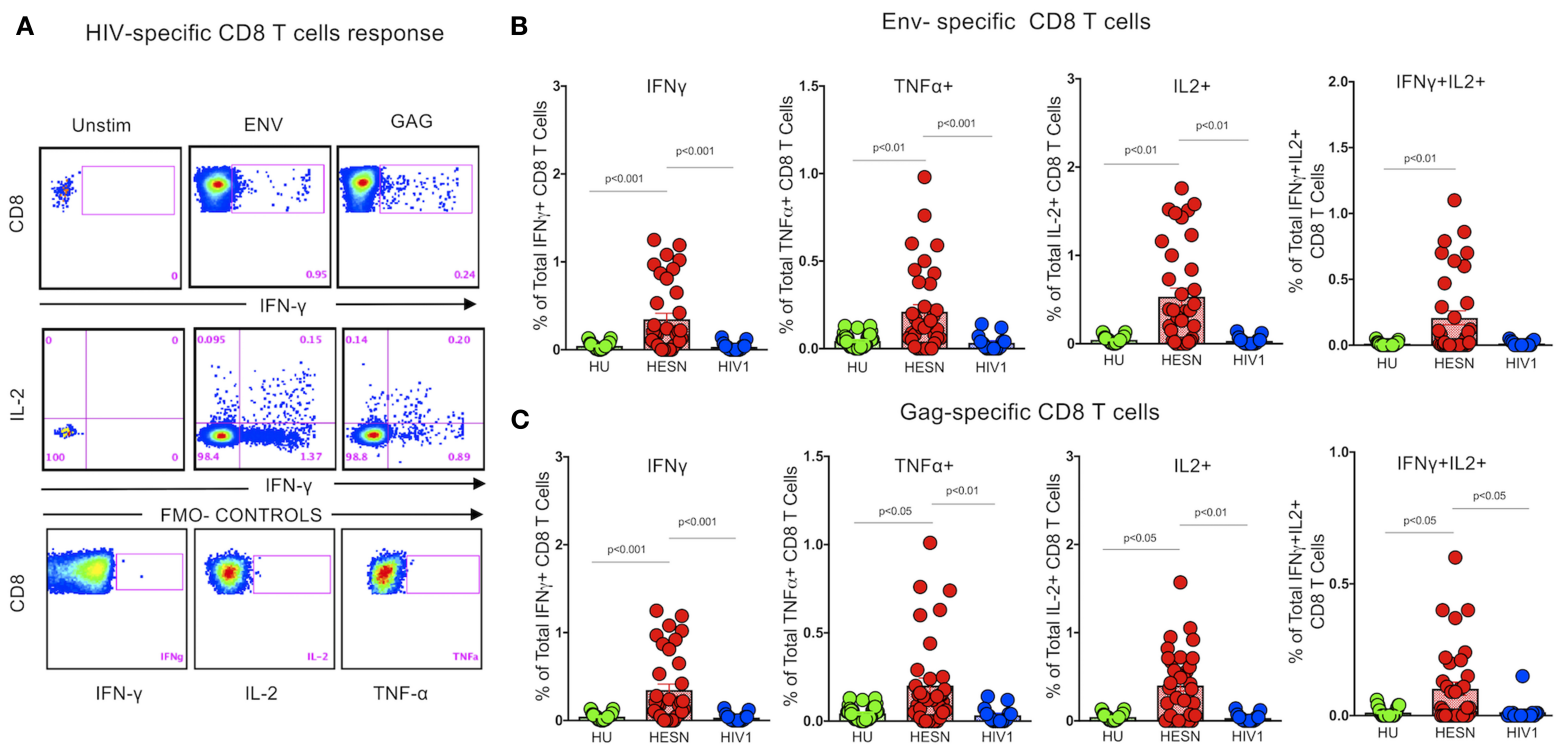
Enrichment of HIV peptide-specific CD8^+^ T cells. Detection of HIV-specific CD8^+^ T cell responses using the ICS assay. Cryopreserved PBMCs from HESN and HU subjects were stimulated with HIV-Pol, -Env, and -Gag peptides or DMSO (negative control) or with SEB (positive control), then stained with surface antibody cocktails, in conjunction with intracellular anti-IFN-γ, TNF-α, and IL-2 antibody, and subjected to analysis by flow cytometry. **(A)** The numbers in each plot denote the percentage of IFN-γ producing CD8^+^ T cells after stimulation, and the percentage of multi-functional IFN-γ^+^ IL-2 producing CD8^+^ T cells after stimulation. **(B)** Percentage of IFN-γ, TNF-α, IL-2, and multi-functional IFN-γ^+^IL-2^+^ Env-specific CD8^+^ T cells in the study groups. **(C)** Percentage of IFN-γ, TNF-α, IL-2, and multi-functional IFN-γ^+^IL-2^+^ Gag-specific CD8^+^ T cells in the study groups. The dot plots represent the median, 25th and 75th percentile and range (IQR). *P-*values were determined using Mann-Whitney's *T-*test. (HESN, HIV exposed seronegative; HIV-1, Progressors; HU, HIV unexposed seronegative controls).

Several studies have reported that HIV-specific poly-functional T cells possibly play a critical role in the non-progression of disease in Elite Controllers (EC) and Long Term Non-progressors (LTNP) (30, 41, 42). Studies have also shown that HIV-1 peptide-specific effector T cells and plasma viral load correlated inversely in HIV-1 progressors (43). We evaluated the proportion of poly-functional HIV-specific CD8^+^ T cells in the HESN group using Intracellular Cytokine Staining assay to identify CD8^+^ T cells with the ability to co-produce cytokines like IFN-γ, TNF-α and IL-2, by stimulating them with a set of HIV-1 exogenous peptides (15-mers overlapping by 11 amino acids) grouped into three pools: HIV-1-Env, -Gag, and -Pol (Figure 3, Supplementary Figure 1). Cells producing multiple cytokines were identified using Boolean gates. The magnitude of the total antigen-specific CD8^+^ T cell response was found to be significantly higher in the HESN group than in the HU group. Multiple cytokines (TNF-α^+^IFN-γ^+^)/(IL-2^+^ IFN-γ^+^) producing CD8^+^ T cells were also predominantly seen only in the HESN group. In contrast, lower levels of poly-functional CD8^+^ T cells were seen in HIV-1 infected individuals (Figures 3A–C). Cumulative analysis of the data demonstrates that HIV-exposed seronegative individuals distinctly possess a more robust HIV-specific CD8^+^ T cell response as compared to HIV-1 progressors and HU controls. Data for the HIV progressor group for this analysis was taken from a previously published study from YRG Care, Chennai (44).

### Efficient CD8^+^ T Cell-Mediated Inhibition of Different Clades of HIV-1 by HESN Individuals

**Figure 4:**
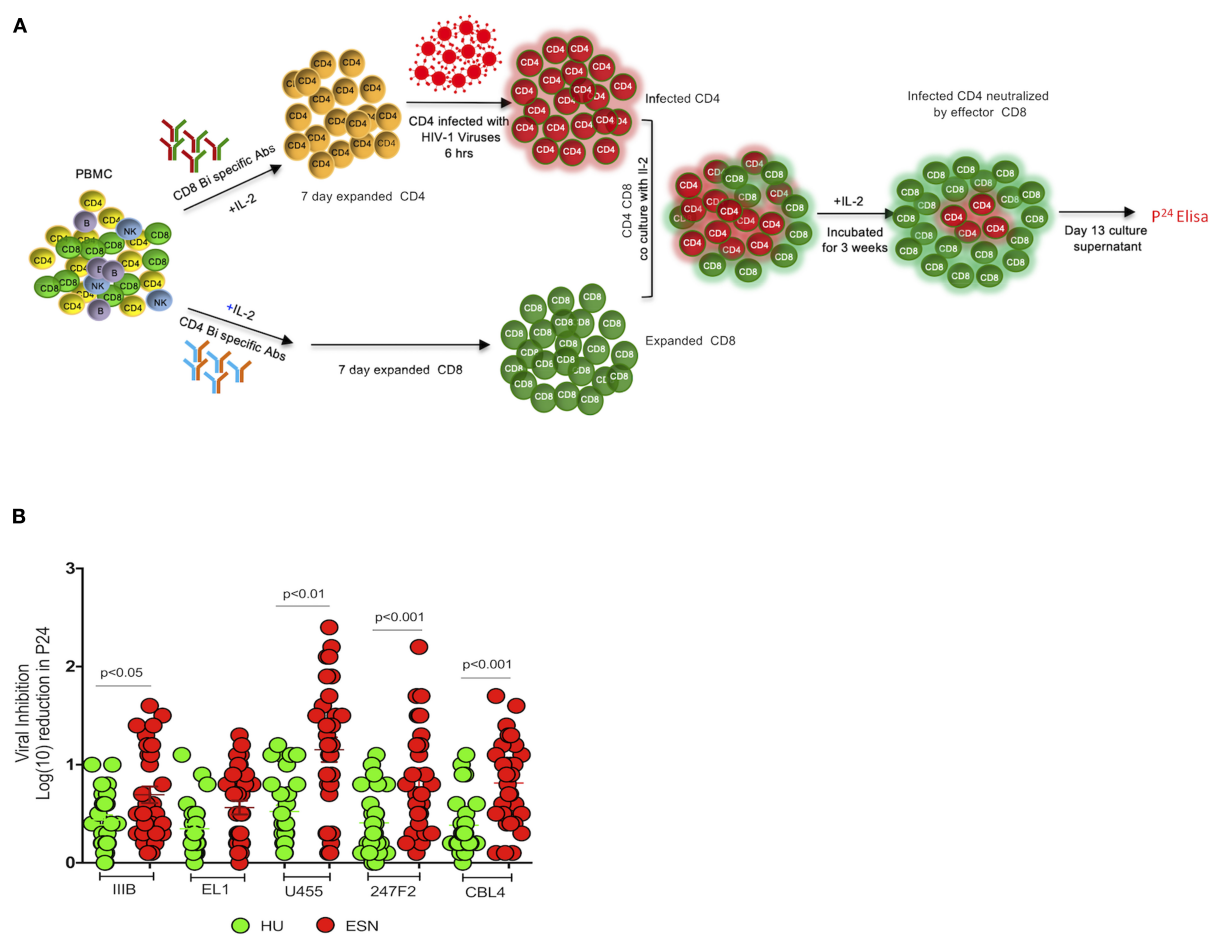
CD8^+^ T cell-mediated inhibition of diverse clades of HIV-1 using viral inhibition assay: **(A)** Schematic diagram of viral inhibition assay. PBMCs were resuspended in Roswell Park Memorial Institute (RPMI) medium with 10% heat-inactivated fetal calf serum to generate ***CD4***^**+**^ ***target and CD8***^**+**^ ***effector T cells;*** 50 U of IL-2 and 0.5 μg/mL of CD3/CD4 or CD3/CD8^+^ bispecific antibodies (Human Immunology laboratory, London) were added, respectively. Culture volumes were doubled at days 3 and 6 by the addition of fresh RPMI medium and IL-2. After 7 days, CD4 T cells were infected with exogenous HIV-1 at a multiplicity of infection (MOI) of 0.01 for 4 h. Following this, expanded CD8^+^ T cells were added to the wells of a tissue culture plate containing CD4^+^ T cells infected with each clade of HIV at a 1:1 ratio and incubated for 15 days. Half of the culture supernatant was replaced with medium containing IL-2 on days 3, 6, 8, and 10. Supernatant p24 content was measured on day 13 by enzyme-linked immunosorbent assay (ELISA) (PerkinElmer). CD8^+^ T cell-mediated inhibition was calculated as the log_10_ reduction in p24 content of day 13 CD8^+^ and CD4^+^ T cell co-culture supernatants compared to that of CD4^+^ T cells alone. **(B)** Viral inhibition activity in terms of log_10_ reduction in p24 levels in HESN and HU groups; Five HIV-1 subtypes were tested: 247FV2 (clade C), CBL4 (clade D), ELI-1 (clade A/D), IIIB (clade B), and U455 (clade A). Graphical representation of the viral inhibition activity in terms of log_10_ reduction in the HESN and HU groups. The box and whiskers plots represent median, 25th, and 75th percentile and range (IQR). *P-*values were determined using Mann-Whitney's *T-*test. (HESN, HIV exposed seronegative; HU, HIV unexposed seronegative controls).

Previous studies have reported that highly proliferative effector CD8^+^ T cells were able to eliminate HIV-infected target cells *in vitro* (45, 46). However, the frequency of these cells did not correlate with *in vivo* virus control. There exists very limited evidence to demonstrate direct T cell-mediated antiviral activity of CD8^+^ T cells on diverse HIV-1 isolates (47–49). We evaluated the breadth of CD8^+^ T cell-mediated antiviral activity in the HESN population using the viral inhibition assay (30). The HIV-1 primary isolates used for this analysis were 247FV2 (clade C), ELI-1 (clade A/D), CBL4 (clade D), U455 (clade A) and IIIB (clade B). HIV-1 p24 antigen levels were measured in culture supernatants of 13-days co-culture of CD8^+^/CD4^+^ cells, and the levels were compared with that of CD4^+^ T cell culture alone. CD8^+^ T cell-mediated inhibition was expressed as log_10_ reduction in p24 content in day 13 cultures (Figure 4A). No viral inhibition activity was detected in the C group, but a significant VIA response was observed in the HESN group. VIA response was evident against four out of five HIV-1 isolates tested in the HESN group. Eleven out of 30 HESN women exhibited VIA activity against U455 (*p* < 0.01), 6 exhibited VIA activity against 247FV2 (*p* < 0.001) and 2 showed VIA activity against IIIB (*p* < 0.05) and CBL-4 (*p* < 0.001), respectively, with log_10_ reduction in p24 levels between 1.5 and 2.4-fold (Figure 4B). None of the HESN women showed VIA activity against ELI-1. These observations indicate that HESN women possess a robust HIV-specific CD8^+^ T cell response that is effective against different HIV-1 clades. CD8^+^ T cell-mediated inhibition was defined as ≥log_10_ reduction in the p24 content of day 13 supernatants from CD8^+^ and CD4^+^ T cell co-cultures as compared to that of CD4^+^ T cells alone. Cut-offs were defined by the 97.5th percentile of the VIA response of baseline samples as estimated using PROC QUANTREG in SAS 9.2 (SAS Institute Inc., Cary, NC). VIA response for each virus was defined as positive if the log_10_ inhibition was > 1.5 for the HIV-1 isolates (34).

### Increased Production of Soluble Cytokines and Chemokines in the HESN Group

**Figure 5:**
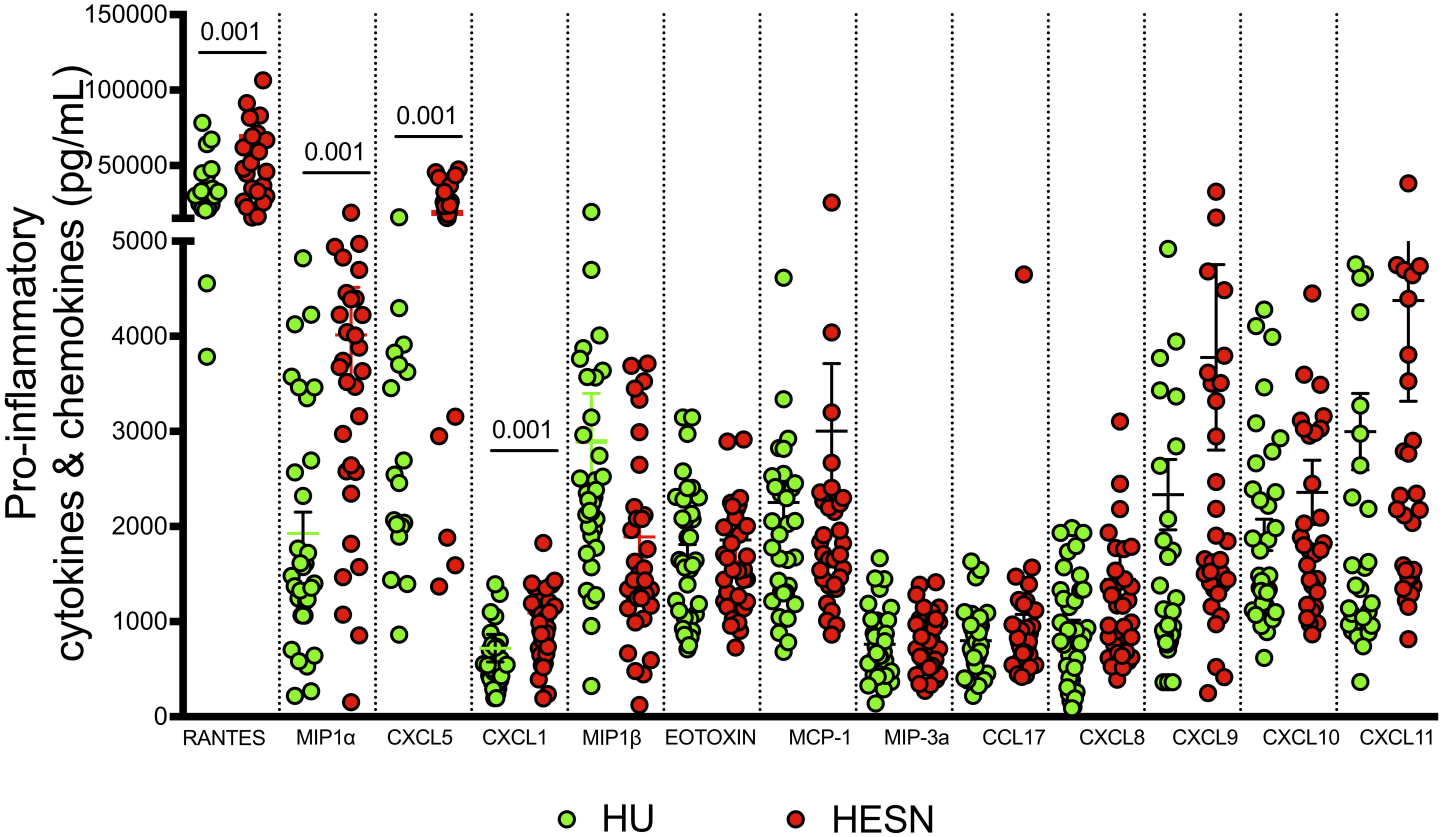
Estimation of pro-inflammatory cytokines and chemokines: Levels of pro-inflammatory cytokines and chemokines were quantified using cytometric bead array among HESN (*N* = 35) and HU (*N* = 35) groups. The data shown is the median level of cytokines and chemokines [RANTES, MIP-α, ENA-78 (CXCL5), GRO-α (CXCL1), and MIP-1β] in plasma (expressed as pg/mL). The scatter dot plots show the median, 25th, and 75th percentiles and range (IQR). *P-*values were determined using Mann-Whitney's *T-*test. (HESN, HIV exposed seronegative; HIV^+^ART^+^, HIV-infected women on ART; HIV^+^ART^−^, HIV-infected women naïve to ART; HU, HIV unexposed seronegative controls).

Studies have identified certain plasma biomarkers to be associated with disease progression in HIV infected individuals (50). Similarly, studies have also identified plasma biomarkers associated with resistance to infection in HESN individuals. Our observations of a good effector T cell response with a large poly-functional HIV-specific CD8^+^ T cell population, prompted us to evaluate the expression of soluble effector molecules in the plasma of these individuals. We used a cytometric bead array to measure levels of IL-8, IP-10, EOTAXIN, TARC, MCP-1, RANTES, MIP-1α, MIG, ENA-78, MIP-3α, GRO-α, I-TAC, and MIP-1β in plasma. We found significantly elevated levels of anti-viral chemokines like RANTES (*p* < 0.001), MIP-α (*p* < 0.001), ENA-78 (*p* = 0.005), GRO-α (*p* = 0.001), and MIP-1β (*p* = 0.003) in the HESN group as compared to the control group (Figure 5, Supplementary Table 2). On the other hand, levels of IL-8, IP-10, EOTAXIN, TARC, MCP-1, MIG, ENA-78, and I-TAC were not similar between the groups. Increased levels of pro-inflammatory chemoattractant in HESN women might favor infiltration of innate immune cells and development of a full-blown T cell effector response capable of controlling HIV infection.

## Discussion

Sexual transmission of HIV accounts for the vast majority of new infections worldwide (51). Susceptibility to HIV infection through sexual transmission is highly variable between individuals. Some individuals, despite multiple and repeated exposures to HIV, remain uninfected and display no evidence of the virus. However, the factors and mechanisms that contribute to protection in these individuals are still not known. It is believed that a combination of factors including genetic factors, innate and adaptive immune cells and effector molecules contribute to protection against infection. Identifying the protective immune signature is key to finding a strategy for controlling HIV and protecting against HIV infection.

Potent and broad CD4^+^ and CD8^+^ T cell responses, as well as neutralizing and non-neutralizing antibody responses, are considered to be important for virological control in HIV infection (42). Earlier studies have demonstrated that CD8^+^ T cell-mediated responses are critical for suppressing virus replication and clearing infected cells from the host (43). Poly-functional T cell responses were later shown to correlate significantly with non-progression of disease in HIV-1 infected LTNPs (52). HIV-specific cytotoxic T cell responses have been reported in individuals exposed to HIV but remaining seronegative (41, 53–56). HIV-infected individuals with antigen-specific CD4^+^ and CD8^+^ T cells that produce multiple cytokines like IFN-γ, TNF-α, and IL-2, have been shown to possess lower viral loads as compared to those with cells that make fewer cytokines (57, 58). Further, studies have found that elite controllers have a higher percentage of effector memory cells and HIV-1-specific TSCM cells, indicating their contribution to HIV-1 resistance (59, 60). An inverse correlation has been observed between the proportion of total CD8^+^ TSCM cells and levels of plasma viremia in untreated HIV-1 infected persons (61).

In the current study, we aimed to evaluate the frequency of anti-viral CD8^+^ T cells, expression of phenotypic and functional makers pertaining to memory differentiation, T stem cell-likeness and follicular homing property, as well as cytokine-producing ability in HESN individuals and compared the data with that seen in ART-naïve and treated HIV-1 infected individuals as well as healthy controls. Our results demonstrate that a subset of effector memory CD8^+^ T cells is enriched and expanded in the HESN group. Interestingly, HESN individuals had higher levels of stem cell-like memory CD8^+^ T cells as compared to the HIV^+^ART^+^, HIV^+^ART^−^, and HU groups. TSCM cells are a special subset of T cells that possess self-renewal capabilities; they can differentiate into mature memory or effector T lymphocytes when stimulated through the T cell receptor (61, 62). Interestingly, the HESN group also possessed increased numbers of follicular homing CD8^+^ T cells as compared to the other groups. Our findings are in agreement with that of a very recent study which showed that HIV-infected individuals with low viral loads had a higher proportion of TSCM cells (63). Subsequent studies also reported elevated levels of effector cells and immature memory HIV-1-specific CD8^+^ T cells in elite controllers. All this evidence suggests a plausible role for HIV-1-specific TSCM cells in HIV-1 resistance (61).

A very recent study showed that TSCM cells are regulated by transcription factor TCF-1 and that HIV and SIV-specific CD8^+^ T cells from natural controllers expressed high levels of TCF-1. TSCM CD8^+^ cells are also known to express high levels of the follicular homing marker, CXCR5 (10). Our study found higher levels of CXCR5 expression on CD8^+^ T cells with effector memory phenotype in HESN individuals. To our knowledge, this is the first study reporting higher frequencies of TSCM, CXCR5^+^ CD8^+^ T cells in HESN cohorts from India. The CXCR5^+^ CD8^+^ T cells in blood are believed to migrate to B cell follicles/germinal centers in the lymph nodes. A very recent study carried out in an animal model showed that CXCR5^+^ CD8^+^ cells represent a unique subset with antiviral activity in animals with chronic SIV infection, suggesting a significant role for these cells in the control of pathogenic SIV infection (39, 63–65). Shen et al. (66) demonstrated that CXCR5^+^ CD8^+^ cells residing in the germinal center follicular area play a key role in reducing the number of virus-infected target cells. The current study also found significantly higher numbers of anti-viral CXCR5^+^ CD8^+^ T cells in the blood of HESN individuals. These findings provide encouraging evidence for some of the previously unidentified correlates of protection against HIV infection, although the mechanisms by which these cells are able to restrict HIV in HESN individuals require further investigation.

In the present study, we also investigated the function of anti-viral CD8^+^ T cells in HESN individuals by comparing them to that of healthy individuals. We evaluated poly-functional CD8^+^ T cell responses using multi-color flow cytometry and observed that the magnitude of the intracellular cytokine response was significantly higher in the HESN group as compared to the HIV-infected and healthy control groups. Gag and Env-specific immune responses were found to be similar in the HESN group, in spite of the presence of higher numbers of Gag-specific CD8^+^ T cells than Env-specific CD8^+^ T cells in these individuals. Similar studies have also reported increased levels of HIV-1 antigen-specific cytokine expression in CD8^+^ T cells as well as CD4^+^ T cells in the HESN, commercial sex workers and LTNPs as compared to progressors and HIV-uninfected healthy controls (26, 55, 67–71). Surprisingly, the HIV-specific cytokine response in the HIV-infected group in our analysis was found to be very low, though other studies like that of Alimonti et al. (70) reported significantly higher levels of IFN-γ in HIV-infected persons as compared to HESN individuals. We presume that the reason for this could be the small sample size in the HIV-infected group (*n* = 10), which actually came from a previously published study carried out in the laboratory of our collaborators (44).

We employed the viral inhibition assay to assess the direct antiviral property of the CD8^+^ T cells in the HESN group. This assay was developed by the Human Immunology Laboratory (HIL) Imperial College, London and employed to detect vaccine-induced CD8^+^ T cell response (34). They define assay positivity as CD8^+^ T cell–mediated inhibition >1.13 log_10_, and inhibition levels lower than this as non-specific and reflecting background activity (34). Others investigators have also observed that CD8^+^ T cells from HIV uninfected subjects mediate a one-log inhibition of HIV-1 replication through non-cytotoxic mechanisms (72–74). We too made similar observations in our laboratory and have therefore used ≥1.5 log_10_ inhibition as the cut-off for defining positive VIA response in an earlier study where we assessed vaccine-induced VIA response (15). As anticipated, CD8^+^ T cell-mediated VIA activity was detected only in HESN women. A total of 8 HESN women showed VIA activity against four of the five virus isolates tested. Put together, the results of the ICS and VIA assays indicate a robust T cell response capable of *in vivo* virus control in HESN individuals.

A deeper understanding of HIV-induced immune activation and the role of the inflammatory response in HIV-1 disease progression remains an important factor in understanding HIV-1 pathogenesis. Higher levels of inflammation have been strongly associated with enhanced HIV-1 replication and disease progression. Elevated levels of serum pro-inflammatory cytokines such as TNF-α, IL-1β, IL-10, CXCL1, and IL-6 are known to increase HIV replication in infected individuals (75). It is therefore obvious that immune activation and an elevated inflammatory response before infection potentially heightens susceptibility to infection and facilitates HIV-1 acquisition (76, 77). In the present study, we found significantly increased levels of RANTES, MIP-1α, MIP-1β, CXCL5, and Gro-α (Growth-regulated alpha protein) in the HESN group. Elevated expression of RANTES, MIP-1α, and MIP-1β suggest that these individuals have robust mechanisms in place to prevent HIV infection at the step of viral entry by blocking the HIV entry co-receptor, CCR5. These markers have been described in association with control of HIV-1 infection by several investigators (78, 79). Induction of CXCL5 and Gro-α possibly suggests prior exposure to HIV in these individuals, as these markers are known to be elevated in HIV-infected individuals (80). RANTES, MIP-1α, MIP-1β, ENA 78, and Gro-α are also known to play a role in adhesion and migration of immune cells and infection control.

To summarize, multiple exposures to the virus in HESN individuals leads to the stimulation and maintenance of an effective immune response capable of containing and/or clearing the virus at the site of infection. The findings of our study strongly suggest that high levels of poly-functional CD8^+^ T cells with stem cell-like and follicular homing characteristics have a significant role to play in the CD8^+^ T cell-mediated control of HIV infection in HESN individuals. Functional characterization of sorted TSCM and Tfh cells would throw light on the mechanistic role played by these cells in the early control of HIV infection. However, this analysis could not be carried out as part of the present study and needs to be pursued in a follow-up study. We thus conclude that a strong CD8^+^ T cell-mediated *ex vivo* viral inhibition activity as well as a protective CD8^+^ T cell response targeting conserved viral epitopes contributes to the early control of HIV infection. We believe that vaccination strategies designed to elicit durable protective immunity should target the generation of these immune cell populations in order to provide effective control of HIV infection.

## Data Availability Statement

The original contributions presented in the study are included in the article/Supplementary Material; further inquiries can be directed to the corresponding author.

## Ethics Statement

The study protocol was approved by the Scientific Advisory Committee of the ICMR-National Institute for Research in Tuberculosis (NIRT), Chennai, India. The study was conducted in accordance with Good Clinical Laboratory Practice (GCLP) guidelines. The study protocol was reviewed and approved by the Institutional Ethics Committee of ICMR-NIRT (IEC ID-2015015) and the Institutional Review Board of the Y. R. Gaitonde Centre for AIDS Research and Education (YRG CARE; YRG-302), Chennai, India. The patients/participants provided their written informed consent to participate in this study.

## Author Contributions

SM, LH, VV, and SSw designed the conceptual framework of the study, designed experiments and wrote the paper. SM performed the experiments and analyzed the data. KT contributed to statistical analyses. TRD, SSa, SP, and KGM contributed to specimen collections. SPT, SSw, AR, JS, SK, UN, and VV contributed to the review and editing of the manuscript. All authors provided valuable input throughout the study.

## Conflict of Interest

The authors declare that the research was conducted in the absence of any commercial or financial relationships that could be construed as a potential conflict of interest.

## Supplementary Material

Supplementary Figure 1 Detection of HIV-1 Pol specific CD8^+^ T cell responses.

Supplementary Table 1 Commercial reagents used for multicolor flow cytometry.

Supplementary Table 2 Dynamics of circulating pro-inflammatory cytokines and chemokines.
