# Supplementary figures and images for: Elevated Numbers of HIV-Specific Poly-Functional CD8+ T Cells With Stem Cell-Like and Follicular Homing Phenotypes in HIV-Exposed Seronegative Individuals
Source: Front Immunol. 2021 Mar 15;12:638144. doi: 10.3389/fimmu.2021.638144 (PMC8056154; doi:10.3389/fimmu.2021.638144)

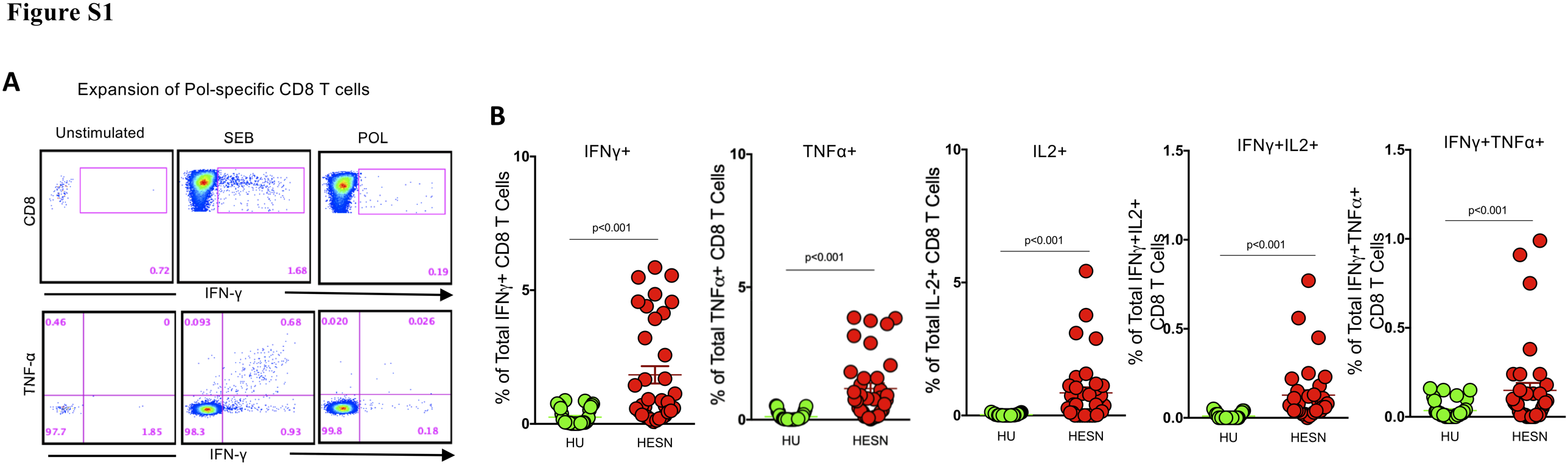

Supplement: Supplementary Figure 1 — Detection of HIV-1 Pol specific CD8+ T cell responses. [file Image_1.TIFF]

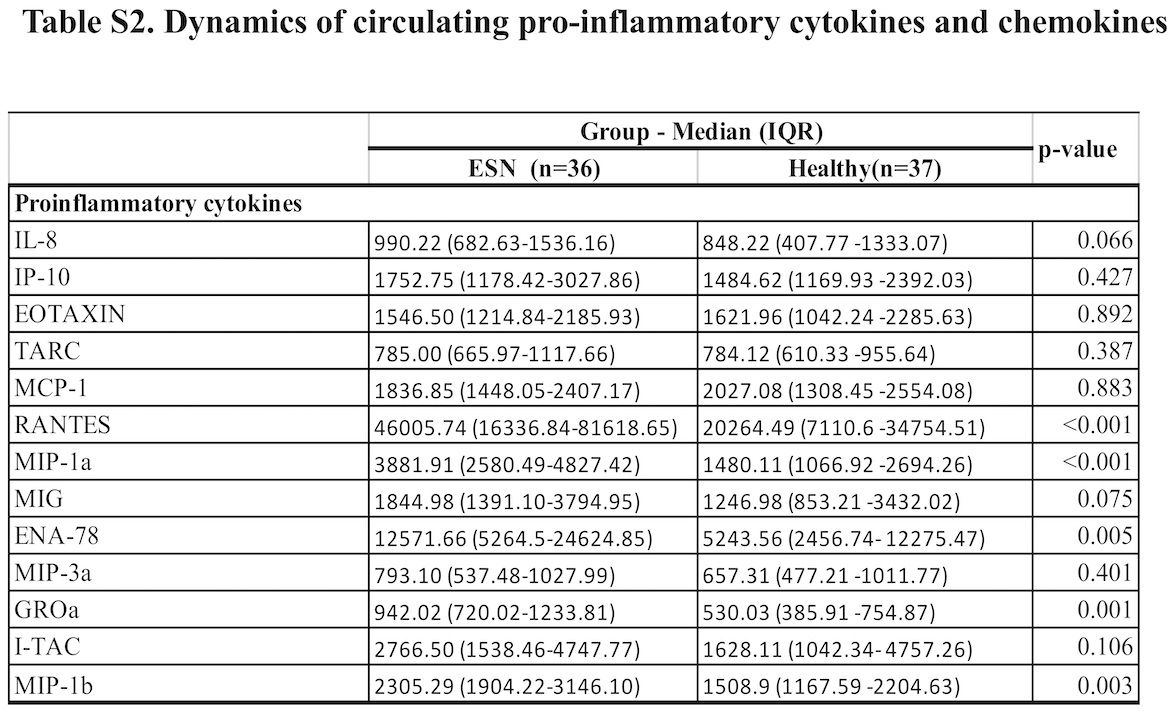

Supplement: Supplementary Table 2 — Dynamics of circulating pro-inflammatory cytokines and chemokines. [file Table_S2.TIFF]
